# An Infrequent Extraintestinal Manifestation of Ulcerative Colitis: Pulmonary Necrobiotic Nodules

**DOI:** 10.7759/cureus.9774

**Published:** 2020-08-16

**Authors:** Adam S Myer, Kushang Shah, Kinner M Patel

**Affiliations:** 1 Internal Medicine, Stony Brook University, State University of New York, Stony Brook, USA; 2 Internal Medicine, Stony Brook Medicine University Hospital, Stony Brook, USA; 3 Pulmonary and Critical Care Medicine, Stony Brook University Hospital, Stony Brook, USA

**Keywords:** ulcerative colitis, inflammatory bowel disease, pulmonary nodules

## Abstract

Pulmonary necrobiotic nodules are a rare extraintestinal manifestation (EIM) of inflammatory bowel disease (IBD), which are often overlooked when diagnosing cavitary pulmonary nodules. We present this case to highlight the importance of a thorough differential diagnosis, which includes EIMs of ulcerative colitis (UC), in this case as necrobiotic nodules. Herein, we present a 25-year-old male patient with a history of poorly controlled UC who presented with fevers, left-sided abdominal pain, and bloody diarrhea. Imaging revealed cavitary pulmonary nodules without an infectious or malignant etiology. Lung biopsy and pathology confirmed a diagnosis consistent with necrobiotic nodules.

## Introduction

The two major forms of inflammatory bowel disease (IBD) are ulcerative colitis (UC) and Crohn's disease (CD). UC is a condition that affects the rectum and colon, characterized by inflammation of the mucosal layer in a continuous fashion [1]. CD can involve any portion of the GI tract, most commonly the ileocecal region, often with normal bowel in between diseased segments. Inflammation can be transmural, resulting in fistula formation.

Extraintestinal manifestations (EIMs) of IBD are seen in 25%-40% of IBD patients [2]. The most common organ systems affected are musculoskeletal, dermatologic, ocular, and hepatobiliary [3]. Pulmonary involvement is rare, and the true prevalence is unknown [4]. Pulmonary manifestations include a wide variety of patterns, such as upper airway (glottis and subglottic edema), large airway (bronchiectasis), small airway (bronchiolitis obliterans syndrome), pulmonary vasculature and thromboembolic disease, and lung parenchymal disease [5]. One pattern of lung parenchymal disease associated with IBD is necrobiotic nodules, which requires a thorough workup because of its resemblance to other autoimmune and infectious etiologies.

## Case presentation

**Figure 1:**
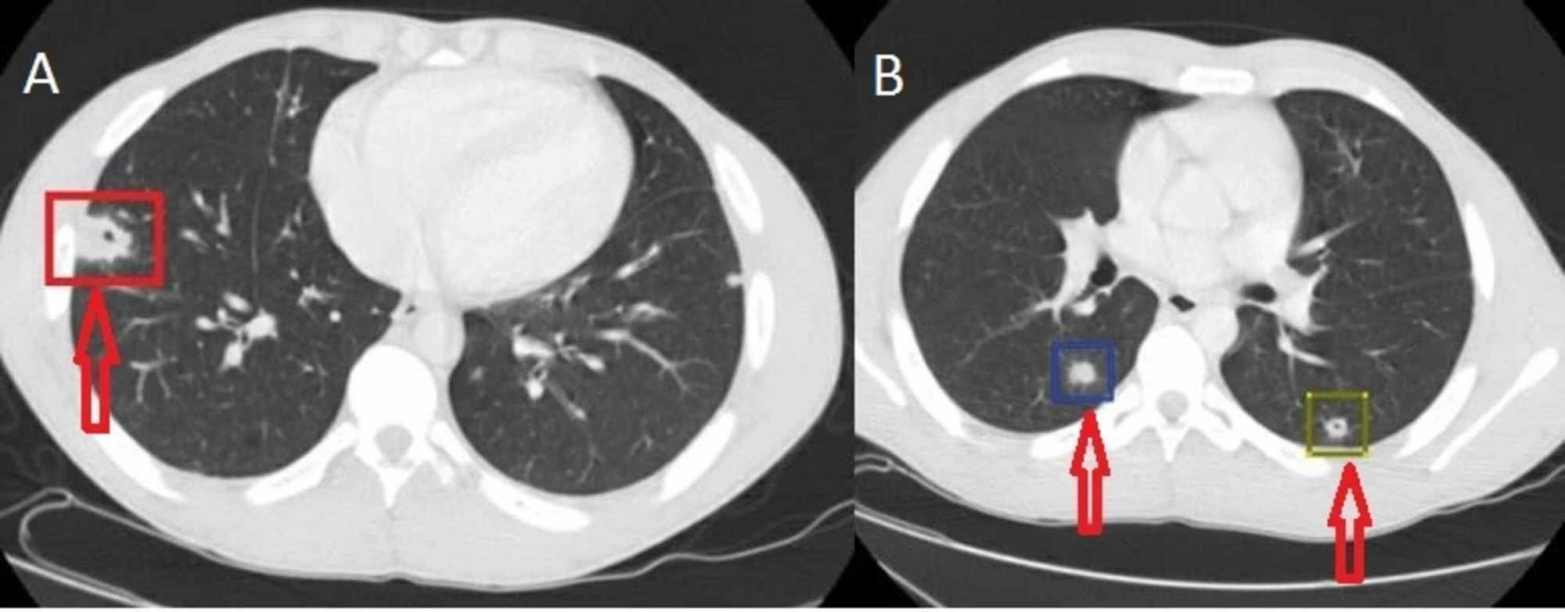
CT of the chest with IV contrast showing several scattered lung nodules with evidence of necrosis and cavitation. The largest nodule (A, red square) is seen in the right lower lobe and does demonstrate necrosis and cavitation, measuring 2 cm. Also seen are several other smaller lung nodules (B, blue and yellow squares).

A 25-year-old male patient with a history of UC presented with fevers, chest soreness, left-sided abdominal pain associated with urgency, and 15-20 episodes per day of bloody diarrhea. CT of the chest, abdomen, and pelvis did not show any evidence of colitis but did reveal multiple necrotic, cavitary pulmonary nodules (Figure 1), and the patient was started on IV vancomycin, cefepime, and Flagyl. The patient was diagnosed with UC two years prior and had been on mesalamine along with steroid suppositories, which he was noncompliant with. The patient denied any history of drug abuse, cardiovascular disease, or dental procedures done in the recent past. Social history was significant for former eight pack-year history of cigarettes smoking and vaping, which he stopped one month prior to presentation.

On admission, the patient was normotensive, afebrile, and saturating >96% on room air. On physical exam, there were coarse breath sounds, tenderness to palpation in bilateral lower quadrants of the abdomen with no rebound tenderness, no cardiac murmurs, Janeway lesions, Osler nodes, or Roth spots. Laboratory markers showed normal chemistry and a hemoglobin of 8.7 g/dL with a mean corpuscular volume (MCV) of 72.9, platelet count of 352 K/µL, white blood cell (WBC) 11.35 K/µL, erythrocyte sedimentation rate (ESR) of 64 mm/hr, C-reactive protein (CRP) of 3.5 mg/dL, and fecal calprotectin 580 µg/g. Infectious etiologies were negative for HIV, hepatitis B, histoplasmosis, coccidioidomycosis, blastomycosis, Coxiella burnetii, Brucella, Clostridium difficile, Aspergillus, and Lyme disease. Acid-fast bacillus (AFB) culture from the sputum revealed no AFB, and stool culture showed no ova and parasite growth.

**Figure 2:**
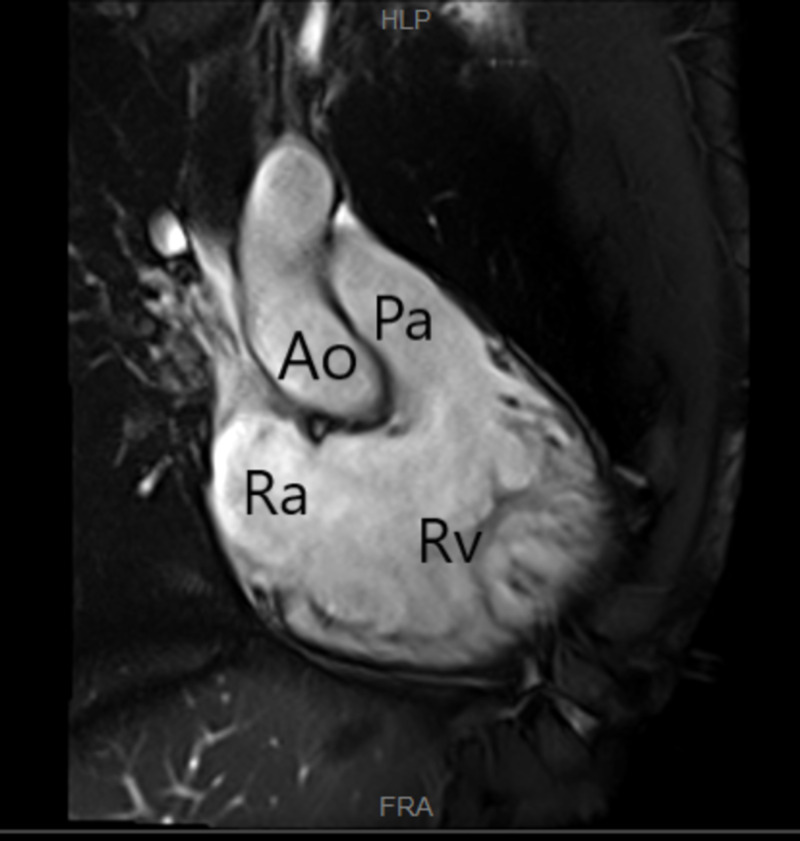
Cardiac MRI white blood imaging, coronal view showing right atrium (Ra), right ventricle (Rv), pulmonary artery (Pa), and aorta (Ao). No evidence of intracavitary thrombus or valvular vegetation.

A transthoracic echocardiogram (TTE) was performed, which showed a possible tissue density on the pulmonic valve. Subsequently, a cardiac MRI (Figure 2) was performed to further characterize the pulmonic valve and did not show any vegetations. Blood cultures showed no growth of organisms. Autoimmune workup for granulomatosis with polyangiitis, microscopic polyangiitis, and rheumatoid arthritis was negative along with negative anti-citrullinated protein antibody (anti-CCP) and rheumatoid factor (RF). Antinuclear antibody (ANA) positivity titer was 1:80, which was deemed to be insignificant in our patient. Antibiotics were discontinued, as the concern for infection remained low.

**Figure 3:**
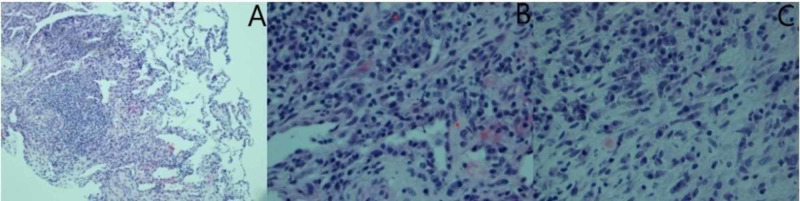
Lung tissue from CT-guided lung biopsy demonstrating fibrosis with mixed inflammatory inflitrate, consisting of macrophages, lymphocytes, scattered neutrophils, and plasma cells. Image A (not zoomed) and images B and C (magnified).

CT-guided lung biopsy was done, which revealed fibrosis with mixed inflammatory infiltrate containing macrophages, lymphocytes, scatter neutrophils, and plasma cells (Figure 3). Mesalamine was stopped as the patient noticed return of abdominal pain with it. Furthermore, case studies have shown pulmonary infiltrates with related 5-aminosalicylic acid derivatives [6]. The patient was treated with 40 mg oral prednisone once a day that was tapered by 10 mg once a week and transitioned to adalimumab. Symptoms gradually improved with therapy. 

## Discussion

Our case describes a rare case of necrobiotic pulmonary nodules in a patient with poorly controlled UC. Necrobiotic nodules are sterile nodules composed of inflammatory cells with necrosis [7]. On imaging, these nodules can appear round and well defined, exhibiting cavitation [5]. The pathophysiology of pulmonary EIM in IBD patients is not entirely known; however, both the respiratory and intestinal mucosa are derived from the primitive foregut. Both epithelia contain submucosal lymphoid tissue, and exposure to antigens via inhalation or ingestion can trigger inflammation. Another hypothesis suggests that lung involvement in IBD can be due to systemic inflammatory mediators released from inflamed bowel mucosa [8].

The appearance of these necrobiotic nodules can mimic other cavitating diseases, making them a diagnostic challenge. Before making this diagnosis, a pertinent, yet thorough differential (Table 1) that includes infectious, autoimmune, vasculitis and malignant etiologies must be excluded. On initial presentation, there was concern for an infectious process causing septic emboli to the lungs. However, the blood cultures revealed no growth and low infectious markers, and unrevealing cardiac MRI ruled out the possibility of an infectious etiology. Necrobiotic nodules may also be seen in rheumatoid arthritis and granulomatosis with polyangiitis; however, RF, anti-CCP, and antineutrophil cytoplasmic antibody (ANCA) were negative [4]. Lung biopsy was then done, which revealed histologic confirmation of necrobiotic nodules.

**Table 1:** Differential diagnosis of cavitating lung nodules.

Differential	Specific Diagnosis
Autoimmune	Rheumatoid arthritis, pyoderma gangrenosum, sarcoidosis, inflammatory bowel disease
Vasculitis	Granulomatosis with polyangiitis, Behcet’s disease
Malignancy	Primary lung (squamous cell carcinoma, adenocarcinoma), lymphoma, metastatic disease
Infectious	Septic emboli, lung abscess (Streptococcus, Staphylococcus, Klebsiella), Mycobacterium tuberculosis, Mycobacterium avium complex, aspergillosis, histoplasmosis, blastomycosis,coccidioidomycosis, paracoccidioidomycosis
Cystic	Langerhans' cell histiocytosis, lymphangioleiomyomatosis, idiopathic pulmonary fibrosis

In review of the literature, there are several case reports of pulmonary necrobiotic nodules in both UC and CD patients; however, the absolute incidence is unknown. Per our review, there are more documented cases of necrobiotic nodules in CD compared to UC. There are two reported cases of pulmonary necrobiotic nodules in UC: a 70-year-old male with UC, who presented with hemoptysis and found to have cavitary pulmonary nodules; biopsy confirmed necrobiotic nodules. The patient was treated with a two-week course of 60 mg prednisone daily and subsequent imaging showed resolution [9]. The second case is of a 17-year-old male diagnosed with UC and primary sclerosing xholangitis (PSC) two years prior in remission, who presented with cough and chest pain. Imaging showed pulmonary nodules, which were biopsied and consistent with necrobiotic nodules. The patient was treated with prednisone and follow-up CT showed greater than 75% reduction of nodule size [10]. There was also a case of a 22-year-old female with UC who had cavitary pulmonary nodules on imaging; however, they were not biopsied [11]. Although the majority of case reports show improvement of pulmonary EIM with steroid therapy, there are no guidelines on duration, dosage, and follow-up imaging [10].

## Conclusions

In IBD patients presenting with pulmonary nodules, it is important to keep necrobiotic nodules as a differential. Etiologies such as autoimmune, infectious, and malignancy should be excluded before coming to this diagnosis. The necrobiotic nodules present in IBD seem to respond to steroid therapy; however, further studies are indicated to determine the duration, dosage, and follow-up testing needed. 
